# Transcript Annotation in FANTOM3: Mouse Gene Catalog Based on Physical cDNAs

**DOI:** 10.1371/journal.pgen.0020062

**Published:** 2006-04-28

**Authors:** Norihiro Maeda, Takeya Kasukawa, Rieko Oyama, Julian Gough, Martin Frith, Pär G Engström, Boris Lenhard, Rajith N Aturaliya, Serge Batalov, Kirk W Beisel, Carol J Bult, Colin F Fletcher, Alistair R. R Forrest, Masaaki Furuno, David Hill, Masayoshi Itoh, Mutsumi Kanamori-Katayama, Shintaro Katayama, Masaru Katoh, Tsugumi Kawashima, John Quackenbush, Timothy Ravasi, Brian Z Ring, Kazuhiro Shibata, Koji Sugiura, Yoichi Takenaka, Rohan D Teasdale, Christine A Wells, Yunxia Zhu, Chikatoshi Kai, Jun Kawai, David A Hume, Piero Carninci, Yoshihide Hayashizaki

## Abstract

The international FANTOM consortium aims to produce a comprehensive picture of the mammalian transcriptome, based upon an extensive cDNA collection and functional annotation of full-length enriched cDNAs. The previous dataset, FANTOM2, comprised 60,770 full-length enriched cDNAs. Functional annotation revealed that this cDNA dataset contained only about half of the estimated number of mouse protein-coding genes, indicating that a number of cDNAs still remained to be collected and identified. To pursue the complete gene catalog that covers all predicted mouse genes, cloning and sequencing of full-length enriched cDNAs has been continued since FANTOM2. In FANTOM3, 42,031 newly isolated cDNAs were subjected to functional annotation, and the annotation of 4,347 FANTOM2 cDNAs was updated. To accomplish accurate functional annotation, we improved our automated annotation pipeline by introducing new coding sequence prediction programs and developed a Web-based annotation interface for simplifying the annotation procedures to reduce manual annotation errors. Automated coding sequence and function prediction was followed with manual curation and review by expert curators. A total of 102,801 full-length enriched mouse cDNAs were annotated. Out of 102,801 transcripts, 56,722 were functionally annotated as protein coding (including partial or truncated transcripts), providing to our knowledge the greatest current coverage of the mouse proteome by full-length cDNAs. The total number of distinct non-protein-coding transcripts increased to 34,030. The FANTOM3 annotation system, consisting of automated computational prediction, manual curation, and final expert curation, facilitated the comprehensive characterization of the mouse transcriptome, and could be applied to the transcriptomes of other species.

## Introduction

The RIKEN Mouse Gene Encyclopedia project was launched with the aim of cloning and sequencing full-length mouse cDNAs. An international annotation consortium (FANTOM) was organized to annotate the collected mouse cDNAs. In FANTOM1, the consortium annotated 21,076 cDNAs with the development of a Web-based annotation interface [1]. In FANTOM2, this interface was extended to be an all online annotation system from remote sites via the Internet, through the Mouse Annotation Teleconference for RIKEN cDNA Sequences (MATRICS). The increased efficiency and throughput was essential in the functional annotation of 60,770 mouse cDNAs [2].

FANTOM1 and FANTOM2 considerably extended our knowledge of the mouse transcriptome, but compared with the number of predicted protein-coding genes from mouse genome sequencing, the cDNA resource covered only half of all predicted genes. Therefore, cDNA collection from a number of novel cellular and tissue sources was continued. In this process, many novel cDNAs derived from distinct genomic loci were fully sequenced. In FANTOM3, these newly sequenced cDNAs were mapped to the mouse genome and subjected to functional annotation. Given the substantial increase in cDNA sequence information in mouse and other mammalian species since FANTOM2, the new annotation process provided the opportunity to update and improve the previous functional annotation of RIKEN cDNAs from FANTOM1 and FANTOM2.

Here we report the development of the new annotation interface and decision pipeline, and the modification of our annotation strategy to accelerate manual annotation. And we also provide functional annotation of 102,801 mouse full-length enriched cDNAs, to our knowledge the largest such dataset.

The result of this functional annotation was shared among FANTOM3 consortium members for further analyses such as protein coding analysis and noncoding RNA (ncRNA) analysis [3].

## Results/Discussion

### Issues Associated with Optimal Online Annotation

The Web-based online annotation system from FANTOM2 was likewise implemented for FANTOM3. This system allowed all curators to annotate transcripts from remote sites around the world through the Internet and resulted in significant acceleration of the manual annotation process. Nevertheless, time remained an issue. Even 10 min spent on manual annotation of each transcript would mean that the total task would consume 15,000 h, and our aim was to complete the task within a matter of weeks. In FANTOM2, curators could enter comments when they encountered problematic cDNAs or ones that were difficult to annotate. However, it was a heavy burden for expert curators, who reviewed and corrected annotations, to read all written comments and correct annotations one by one. For these and other reasons, we introduced a precomputational pipeline in which the annotator could accept the automated decision by ticking a series of boxes. Only where there was some ambiguity, or a better alternative name, was the annotator required to assess additional data and enter alternative decisions. In general, this process reduced the annotation time for unequivocal cases down to 10–20 s.

### Modification of Annotation Rules and Pipeline for FANTOM3

We updated the original annotation rules that were determined during the FANTOM2 meeting [2,4,5] in order to improve the quality of curation, and introduced these into the automated annotation pipeline. The annotation items in the FANTOM3 new rule set are summarized in Table 1.

**Table 1:**
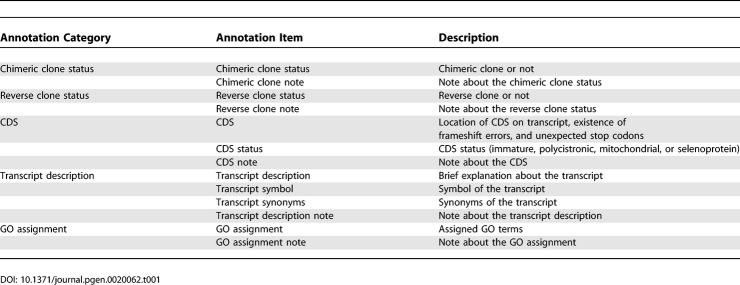
Annotation Items in FANTOM3

Firstly, coding sequence (CDS) annotation items were expanded in FANTOM3. In FANTOM2, curators annotated the following four items: CDS status (CDS region for coding, UTR region only, or no CDS), completeness of 5′ and 3′ ends of CDS, maturity of transcripts, and presence of in-frame insertion/deletion errors and stop codons. In FANTOM3, three additional items were introduced: exact positions of in-frame insertion/deletion errors, and flags for selenoproteins and mitochondrial transcripts with unique codon usage. This information was used for computational translation to make a complete dataset of protein-coding transcripts, and it allowed us to avoid unwanted frameshifts and stop codons in the middle of a CDS region.

Secondly, the set of CDS prediction algorithms, the outcomes of which are displayed at the top level to the annotator, was changed based upon our previous experience. ProCrest (unpublished) and NCBI CDS Predictor (unpublished), which were used in FANTOM2, were phased out because they cannot identify exact positions of in-frame insertion/deletion errors, although they are able to predict whether these errors exist or not within CDS regions [4]. Instead, three other algorithms were introduced: CRITICA [6], mTRANS (M. Furuno, unpublished data), and CombinerCDS [7]. In FANTOM3, curators could make their judgment on a CDS region by comparing all of the CDS predictions from DECODER [8], rsCDS [5], longest ORF, truncated longest ORF, CRITICA, mTRANS, CombinerCDS, and FANTOM2 curation (for FANTOM2 cDNAs).

**Figure 1:**
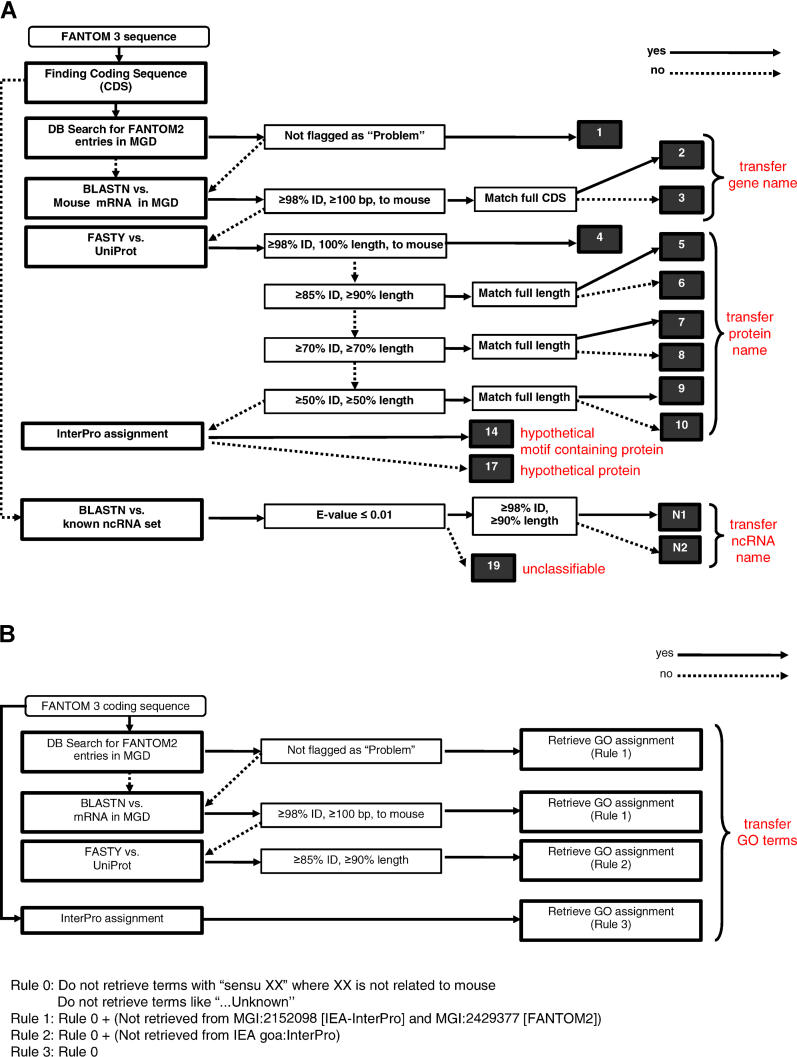
Annotation Pipelines for Transcript Description and for GO Terms (A) Pipeline for transcript description. Query sequences falling into categories (black boxes) 1–3 were assigned the description of the matched target sequence DNA entry in MGI symbols, and synonyms were also transferred to our annotation database. Queries falling into categories 4–10 were assigned a transcript description corresponding to the matched protein name. For query sequences falling into category 5 or 6, the keyword “homolog” was appended to the matching protein name. Sequences assigned to category 7 or 8 were denoted with the prefix “similar to” attached to the target sequence name. The prefix “weakly similar” was used to identify sequences assigned to category 9 or 10. For all sequences in categories 5–10, the name of the organism corresponding to the matched protein was appended to the assigned transcript description. If a query was assigned to category 14, its transcript description was “hypothetical [InterPro domain name] containing protein.” Query sequences assigned to category 17 and 19 were annotated as “hypothetical protein” and “unclassifiable,” respectively. Query sequences grouped into category N1 or N2 were assigned the description of the matched target ncRNA entry. For query sequences falling into category N2, the keyword “homolog of” was appended to the matching ncRNA name. (B) Pipeline for GO terms. DB, database.

Thirdly, we improved our annotation pipelines for assigning transcript descriptions (renamed from “gene names” in FANTOM2), symbols (renamed from “gene symbols”), and synonyms to transcripts (Figure 1A) and for assigning Gene Ontology (GO) terms (Figure 1B). We excluded some protein motif detections and transcript clustering procedures from our previous pipelines, making it possible to simplify the automation. Instead, one new step was introduced to identify known ncRNAs. When a transcript has no coding regions and its sequence significantly matches against a known ncRNA set retrieved from RNAdb [9], the name of its known ncRNA is transferred as its transcript description. Otherwise, the transcript description of the ncRNA becomes “unclassifiable.” As for the GO assignment pipeline, the most significant match was searched in the order of directly assigned Mouse Genome Informatics (MGI) markers, DNA matches, and protein matches, and then its GO assignments were transferred to the query transcript. If InterPro motifs were detected in transcripts, GO assignments on the motifs were also transferred and combined with ones for a significant match.

Fourthly, new annotation items to identify problematic clones were added. In the FANTOM3 annotation system, two buttons for these problematic clones, chimeric clones and reverse clones, were introduced to simplify the annotation process. If a cDNA is deemed to be derived from two or more mRNAs or to be a contaminant from *Escherichia coli,* it is curated as “chimeric clone.” If a cDNA has evidence that implies cloning in the reverse direction, for example, having CT-AC splicing patterns rather than GT-AG ones, it is curated as “reverse clone.” These problematic entries are then automatically excluded from further curation and analyses.

### Modification of the Curation Interface for FANTOM3

To help curators annotate accurately, the curation interface was improved from that of FANTOM2. Information such as MGI assignment, cDNA status prediction, sequence quality, expressed sequence tag mapping, genome mapping, splicing information, predicted transmembrane regions, and protein motifs was provided on the curation screen. Some information was provided in a simple graphical display to expedite rapid decisions. Moreover, additional information such as raw alignments and hyperlinks to public databases could be accessed by clicking corresponding bars in the cDNA summary image section.

In the new FANTOM3 interface, annotators were provided with an initial computational annotation that the curators were then required to accept or reject by clicking buttons. To simplify the annotation process when the computational annotation was rejected, several major reject reasons and alternative CDS predictions, CDS statuses, transcript descriptions, and GO terms were provided as a list with checkboxes, and the curators were prompted to select an appropriate one. Curators were also encouraged to add notes on each transcript, based upon their background knowledge.

### The Annotation Process

The computational annotation in FANTOM3 was carried out prior to manual annotation, as in FANTOM2. The FANTOM3 annotation pipelines for assigning transcript descriptions and for GO assignments are summarized in Figure 1A and 1B, respectively. Subsequent manual annotation was carried out sequentially. The potential protein-coding transcripts, whose predicted CDS regions were longer than 100 amino acids in length based upon the computational prediction, were annotated first because these require the least input and are of greatest interest to the scientific community. Out of 30,476 FANTOM3 potential coding transcripts, 20,027 (65.7%) initial computational annotations were manually accepted, and 6,997 (23.0%) transcripts were easily annotated by choosing the alternatives provided. Thus, more than 85% of coding transcripts were easily annotated by just clicking buttons on our annotation interface, indicating that the button-based interface indeed contributed to accelerated manual annotation for potential protein-coding transcripts.

After manual curation on potential protein-coding transcripts, we next considered annotating potential non-protein-coding transcripts. To reduce human annotation errors, potential non-protein-coding transcripts were classified into several subcategories and were released stepwise depending on their coding potential. The transcripts that completely or partially matched known genes at the DNA level were open to curators first, followed by the transcripts that showed similarity to known genes at the amino acid level. Finally, the transcripts that were just covered with expressed sequence tags were subjected to manual curation. Out of 11,555 potential non-protein-coding transcripts, 7,343 (63.5%) transcripts were annotated as non-protein-coding. And 1,893 (16.4%) and 386 (3.3%) transcripts were annotated as immature and truncated forms, respectively.

### Review of Functional Annotation

To improve the quality of the functional annotation dataset, a review process was carried out following the manual curation. Expert curators were selected from all registered curators based on their performance, and they reviewed the rejected entries. In FANTOM3, computational filtration was intensively performed to lighten the burden for expert curators. Several criteria are discussed below.

In eukaryotes, nonsense-mediated mRNA decay is known as a mRNA surveillance mechanism (reviewed in [10,11]). It has been recently reported that some mRNAs that have premature termination codons are not degraded by the nonsense-mediated mRNA decay mechanism, and that the “50 nucleotide rule” of nonsense-mediated mRNA decay cannot always be applied to the evaluation of annotation results. However, this “50 nucleotide rule” was useful for a rough screening to extract the transcripts that might be incorrectly annotated. In the FANTOM3 review process, cDNA entries for which the 3′ end of the curated CDS was 50 nucleotides or more upstream of the 3′-most exon/intron junction were computationally extracted. These cDNA entries were intensively reviewed, and apparent misannotations were corrected by expert curators.

Flanking adenine-rich sequence at the 3′ end of a transcript suggests the possibility that the cDNA could be produced by internal priming of oligo-dT primer. Therefore, we extracted the transcripts that had more than ten adenosines in the 20 flanking nucleotides by using mouse genome sequence, and these transcripts were manually reviewed by expert curators. If transcripts seemed to be produced by internal priming of coding transcripts, they were curated as coding/immature.

In FANTOM3, we also developed a genomic element browser by customizing the generic genome browser [12,13] to review annotations based on their genomic loci. In this browser, all FANTOM transcripts are aligned on their genomic loci, accompanied by information on annotation (e.g., curated coding region, gene name, coding/noncoding, clone ID, and strand orientation). This browser allowed the expert curators to compare all transcripts that were located at the same loci and to correct annotation when necessary.

### Conclusions

In FANTOM3, 42,031 transcripts were newly annotated and the functional annotation of 4,347 FANTOM2 transcripts was updated with the improved annotation system. Combining the results of FANTOM2 and FANTOM3, 102,801 cDNAs were functionally annotated by the international effort. Out of these, 47,761 and 8,961 transcripts were annotated as complete coding and truncated coding, respectively, and 34,030 transcripts were annotated as non-protein-coding (Table 2).

**Table 2:**
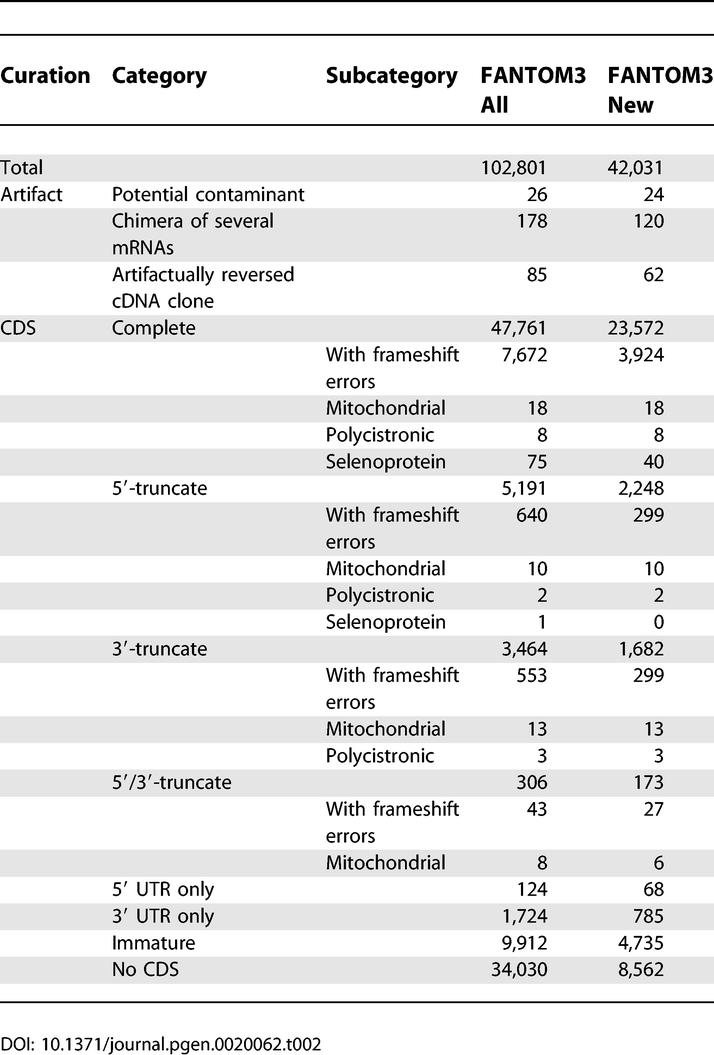
Summary of Annotation in FANTOM3

Our FANTOM3 annotation system largely contributed to the prompt and precise annotation that was accomplished, and this system could be a model for other mammalian transcriptome projects.

The curated annotation data are available at http://fantom3.gsc.riken.jp/db and ftp://fantom3.gsc.riken.jp/fantomdb/3.0.

## Materials and Methods

### Sequence set and annotation.

We annotated 102,801 sequences derived from RIKEN mouse full-length enriched cDNA libraries [3]. The set consists of 60,770 FANTOM2 [2,14] and 42,031 novel isolated sequences. Of the 60,770 FANTOM2 sequences, 932 were updated after the FANTOM2 meeting. For the FANTOM2 sequences that were not curated during the FANTOM3 period, the gene names assigned in FANTOM2 were transferred as their transcript descriptions.

### Mapping of transcript sequences to the mouse genome.

Transcript sequences were mapped to the mouse genome (assembly mm5) in several stages. In the first stage, the sequences were aligned to the genome using BLAT version 30 [15] with options –ooc = 11.ooc –fine –q = rna. Low-quality alignments were then removed using pslReps (distributed with BLAT; http://www.soe.ucsc.edu/~kent/exe/linux/blatSuite.zip) with options –minAli = 0.96 –nearTop = 0.005. Next, the alignments were post-processed by an algorithm designed to extend transcript-to-genome alignments by using information about exon positions from neighboring alignments (P. G. Engström and B. Lenhard, unpublished data). Subsequently, the highest-scoring alignment or alignments, according to the following formula, were retained for each transcript: round (20,000 × identity + 100 × coverage + 2 × number of introns), where identity = number of matches/(number of matches + number of mismatches + number of non-intron gaps), coverage = number of matches/transcript sequence size, and introns are gaps of at least 20 bp in the transcript sequence only. Ties were broken in favor of assembled chromosomes over unassembled genomic sequence. If there were still two highest-scoring alignments for a transcript, both were displayed in the annotation interface. Finally, adjacent alignment blocks were connected if they appeared to belong to the same exon. The criteria for deciding that blocks belonged to the same exon were adopted from the Sim4 program [16]: (1) gap lengths of less than 50 bp and (2) differences in gap lengths between genome and cDNA sequences of less than 9 bp. In merging blocks, gapped regions were aligned with the stretcher program in the EMBOSS package [17].

### Computational analysis for data preparation.

Assembled full-length cDNA sequences were first masked using RepeatMasker (http://repeatmasker.org) to exclude regions containing known repetitive sequences. FANTOM3 query sequences were searched against mouse non-expressed-sequence-tag mRNA sequences in the MGI database [18] (http://www.informatics.jax.org), against the mouse sequences in dbEST [19] (http://www.ncbi.nih.gov/dbEST), and against known ncRNA sequences in RNAdb (http://research.imb.uq.edu.au/rnadb) [9]. DNA searches were performed using BLASTN [20] with the –F option, which turns off filtering of the query sequences, for the MGI and dbEST database searches and with the default option for RNAdb searches. Protein databases were searched using the FASTY program [21] in the FASTA3 package. The FANTOM3 sequences were searched against the UniProt database [22]. Open reading frames in the cDNA sequences were predicted using DECODER, and those with predicted CDS regions were subjected to an InterPro motif prediction analysis. InterProScan was used to search the InterPro database [23] (http://www.ebi.ac.uk/interpro).

### Coding potential classification.

We flagged sequences “with coding potential” when at least one of the following conditions was satisfied: a protein was matched with greater than 50% identity and greater than 50% length of the target protein, a named InterPro domain was found in the predicted protein sequence, a transmembrane region was detected with the TMHMM program [24], a coiled coil region was predicted with the NCOILS program, a signal peptide was identified with the SignalP program [25], or a CDS longer than 100 amino acids was predicted.

### Annotation pipeline programs and curation interface.

The annotation pipeline programs were implemented as a Perl script that evaluated the evidence at each stage in the process and made a decision at each stage, writing the appropriate annotation to the database using the appropriate controlled vocabulary terms.

The cDNA annotation (curation) interface was implemented as a Web-based application using mod_perl and the gd graphics library on a Linux system running an Apache 2.0 server. All curated annotations and annotation histories were stored in a custom database implemented in a Sybase (http://www.sybase.com) relational database management system. Other data such as similarity search alignments and clone sequences were stored in indexed flat files. 

## Abbreviations

CDS: coding sequence
GO: Gene Ontology
MGI: Mouse Genome Informatics
ncRNA: noncoding RNA
